# The Book World of Medicine and Science

**Published:** 1895-11-30

**Authors:** 


### THE BOOK WORLD OF MEDICINE AND SCIENCE.

DISEASES OF THE UPPER RESPIRATORY TRACT, THE NOSE, PHARYNX, AND LARYNX. By P. Watson Williams, M.D. Illustrated. (Bristol: John Wright and Co.; London: Simpkin, Marshall, Hamilton, Kent, and Co. (Limited); Hillsfield Bros., 82, High Holborn. 1894.)

The author of this work has admirably succeeded in giving within the small compass of 282 pages a succinct and complete outline of the modern views on the pathology and treatment of the affections of the upper respiratory tract. In his preface he states that his manual was intended to supply a want he had long felt in his capacity as a teacher of clinical medicine, viz., an introduction to the study of diseases of the upper air passages that might serve as a practical guide to students and practitioners, and as a stepping stone to the larger and more comprehensive works whose very wealth of detail renders them less suitable for beginners. That such a want existed will hardly be denied by anybody well acquainted with the special literature of the subject; whilst plenty of larger and excellent works of English and American origin exist, most of the small primers and manuals for the use of advanced students devoted to the study of the same diseases are either lamentably incomplete, or utterly unscientific, or full of mistakes, or devoted to practical exigencies only, leaving all theoretical questions aside. The work now under consideration avoids all these drawbacks, and this is no small praise. The author does not claim much originality of views, nor does he attempt to take part in the controversies which are so rife in this particular territory; but, on the other hand, he has formed on all questions connected with his subject a clear judgment of his own, and by expressing this in a form equally lucid and moderate proves himself to be a trustworthy guide for the beginner. Special commendation is due to his extensive knowledge of the literature of his subject, and to the recognition he accords to the achievements of other investigators. His treatment, too, in every respect is thoroughly modern, without at the same time falling into the fault of "polypragmasia." He never forgets that he is a physician, and that local treatment, valuable as it is, is not all in successfully dealing with affections of the upper respiratory tract. In short, the work in every respect admirably accomplishes its purpose. Needless to say, it would be easy to mention a few points in which the views of the reviewer do not completely coincide with those of the author; but instead of picking holes in a conscientious and admirable work of this kind, we prefer to express our pleasure that such a work has been written, and we tender our sincere congratulations to the author.

